# Human foreskin-derived dermal stem/progenitor cell-conditioned medium combined with hyaluronic acid promotes extracellular matrix regeneration in diabetic wounds

**DOI:** 10.1186/s13287-020-02116-5

**Published:** 2021-01-09

**Authors:** Yu Xin, Peng Xu, Xiangsheng Wang, Yunsheng Chen, Zheng Zhang, Yixin Zhang

**Affiliations:** 1 Department of Plastic and Reconstructive Surgery, Shanghai 9th People’s Hospital, Shanghai Jiao Tong University School of Medicine, 639 Zhi Zao Ju Road, Shanghai, 200011 China; 2 Shanghai Tissue Engineering Key Laboratory, Shanghai Jiao Tong University School of Medicine, Shanghai, 200011 China

**Keywords:** Human foreskin, Dermal stem/progenitor cells, Conditioned medium, Diabetic wound healing, Extracellular matrix

## Abstract

**Background:**

Diabetic wounds remain a challenging clinical problem, which requires further treatment development. Published data showed that dermis-derived stem/progenitor cells (DSPCs) display superior wound healing in vitro. The beneficial effects of DSPCs are mediated through paracrine secretion, which can be obtained from conditioned medium (CM). Hyaluronic acid (HA) is especially suitable for skin regeneration and delivering bioactive molecules in CM. This study investigated the effect of human foreskin-derived dermal stem/progenitor cell (hFDSPC)-CM combined with HA on a diabetic mouse model and relevant mechanism in vitro.

**Methods:**

hFDSPCs and human adipose-derived stem cells (hADSCs) were identified, and the respective CM was prepared. PBS, HA, hFDSPC-CM combined with HA, or hADSC-CM combined with HA was topically applied to mice. HE, CD31, CD68, CD86, and CD206 staining was performed to evaluate gross wound condition, angiogenesis, and inflammation, respectively. Masson and Picrosirius red staining was performed to evaluate collagen deposition and maturation. The effects of hFDSPC-CM and hADSC-CM on human keratinocyte cells (HaCaT) and fibroblasts were evaluated in vitro using CCK-8 and EdU assays to determine cell viability and proliferation, respectively. The scratch assay was performed to evaluate cell migration. Tube formation assay was performed on human umbilical vein endothelial cells (HUVECs) to confirm angiogenesis. Extracellular matrix (ECM) metabolic balance-related genes and proteins, such as collagen I (COL 1), collagen III (COL 3), fibronectin (FN), α-SMA, matrix metalloproteinases 1 (MMP-1), matrix metalloproteinases 3 (MMP-3), and transforming growth factor-beta 1 (TGF-β1), were analysed.

**Results:**

hFDSPC-CM combined with HA showed superior wound closure rate over hADSC-CM. Histologically, the hFDSPC-CM combined with HA group showed significantly improved re-epithelialisation, angiogenesis, anti-inflammation, collagen regeneration, and maturation compared to hADSC-CM combined with HA group. In vitro assays revealed that hFDSPC-CM displayed significant advantages on cell proliferation, migration, and ECM regeneration through a TGF-β/Smad signalling pathway compared with hADSC-CM.

**Conclusions:**

hFDSPC-CM combined with HA was superior for treating diabetic wounds. The underlying mechanism may promote proliferation and migration of epidermal cells with fibroblasts, thus leading to ECM deposition and remodelling. Reduced inflammation may be due to the above-mentioned mechanism.

**Supplementary Information:**

The online version contains supplementary material available at 10.1186/s13287-020-02116-5.

## Background

Diabetic wounds remain a challenging clinical problem and are characterised by deficient chemokine production and angiogenesis, decreased fibroblast migration and proliferation, and an abnormal inflammatory response [1, 2]. Current treatment for diabetic wounds include customised dressings, surgical debridement, negative pressure wound therapy, antibiotics, and hyperbaric oxygen [3–5], without targeting the underlying pathophysiology, leading to treatment failure [4].

Although recent studies showed that mesenchymal stem cells (MSCs) isolated from the adipose tissue, bone marrow, umbilical cord blood, and skin exhibited similarity and have been proven to accelerate diabetic wound healing, they have specific features from distinct niches [6–11]. Zomer et al. revealed a greater in vitro wound closure capacity of dermis-derived MSCs than adipose-derived MSCs, showing that dermis-derived MSCs are unique for skin wound healing [12]. Particularly, as a discarded waste, the human foreskin exhibited attractive potential as an abundant source of MSCs for clinical application without ethical concerns [13].

Notably, the beneficial effects of MSCs are mediated through paracrine secretory mechanisms, which are called “secretomes” and mainly consist of various growth factors, microRNA, proteasomes, and extracellular vesicles (EVs). These are more suitable for clinical applications than cell therapy because they circumvent many safety concerns associated with cell therapy. Extracellular vesicles showed good outcomes for diabetic wounds, but the tedious processes of EV preparation limit their application. In contrast, conditioned medium (CM) from MSCs exhibits many merits over EVs, such as being cheaper and quickly obtained, and may yield either equipotent or more potent preparation for clinical application [14]. In addition, skin wound dressings also play crucial roles in improving wound healing. Among which, hyaluronic acid (HA), as a kind of off the shelf biocompatible polymer, is especially suitable for skin regeneration, due to its natural existence in skin tissue and the ability to locally deliver entrapped bioactive molecules [15]. In addition, therapies that integrated HA with other components such as exosomes and silver particles exhibited enhanced healing effects [16, 17]. And HA-based wound fillers can be enriched with further bioactive components, such as conditioned media.

To date, some foreskin-derived fibroblast products have been commercially available due to their pro-healing effect for wound healing, such as Dermagraft® (Organogenesis, Inc.) and Apligraf® (Organogenesis, Inc.). However, the effects of administering human foreskin-derived dermal stem/progenitor cell (hFDSPC)-conditioned medium (hFDSPC-CM) combined with HA on a diabetic mouse model remain uncertain. To this end, hFDSPC-CM with HA was prepared and topically applied to diabetic mice. Human adipose-derived stem cell-conditioned medium (hADSC-CM) was selected as the positive control, which is proven to have obvious therapeutic effect on this disease [18]. In vitro assays were further performed to illuminate the corresponding mechanism of hFDSPC-CM in promoting skin wound healing.

## Methods

### Cell isolation and culture

All tissue samples were collected from Shanghai Ninth People’s Hospital, with written consent obtained from the patients for experimentation prior to surgery. The study was approved by the Ethics Committee of Shanghai Jiao Tong University School of Medicine.

For hADSC isolation, human adipose tissues, obtained from 9 healthy female donors (age range 23–38 years) who underwent liposuction (informed consent was obtained from donors for using adipose tissues in experiment), were mixed as a cell pool, rinsed with saline, and separated from blood vessels and excess fat. Then, samples were digested with collagenase type I (Sigma-Aldrich, St. Louis, MO, USA) for 1–2 h at 37 °C. The samples were then filtered through a 70-μm cell strainer (BD Biosciences, Mississauga, Canada) and mixed with low-glucose Dulbecco’s modified Eagle’s medium (DMEM, Gibco, NY, USA) supplemented with 10% foetal bovine serum (FBS, Gibco, NY, USA) and 1% antibiotics (penicillin 100 U/ml, streptomycin 100 U/ml, Gibco), and then centrifuged at 300×*g* for 10 min. The supernatant and oil drop were discarded. The precipitated cells were resuspended in DMEM, supplemented with 10% FBS and 1% antibiotics, and then cultured in a humidified incubator at 37 °C with 5% CO2. The culture medium was changed every 2 days. Passage 0 to 3 cells were used in the following experiments.

For hFDSPC isolation, human foreskin tissue was obtained from 9 children (age range 2–7 years) undergoing circumcision (informed consent was obtained from the parents of the children for using foreskin tissue in experiment) and mixed as a cell pool. To separate dermis from epidermis, the donated tissues were harvested, minced, and immersed in Dispase II (Roche Applied Science, Indianapolis, IN, USA) overnight at 4 °C. The remaining dermis was separately minced into small pieces followed by digestion with 0.2% collagenase type IV (Sigma-Aldrich) for 2–3 h. The digested cells were resuspended in minimum Eagle’s medium (Gibco, Ontario, Canada) supplemented with 10% FBS and 1% antibiotics. Cells were seeded on tissue culture plates at 1 × 10^3^ cells/cm^2^ and cultured for 24 h, and then washed with phosphate-buffered saline (PBS, Gibco, NY, USA) to remove residual non-adherent cells. After 5 days, the adherent cells were selected for superior colonies in form and quantity for collection and passage. Cells at passages 0 to 3 were used in the following experiments.

For in vitro mechanistic studies, excess skin tissues obtained from 5 female donors (age range 26–36 years; informed consent was obtained from the donors) undergoing plastic surgery were mixed as a cell pool for human fibroblast isolation. The tissues were processed within 2 h post-surgical excision. Cell isolation and culture methods were in accordance with a previous study [19]. The third passage cells were used in the following experiments.

Human immortal keratinocyte cells (HaCaT) and human vascular endothelial cells (HUVECs) were purchased from the American Type Culture Collection (ATCC, Rockville, MD, USA) and maintained in DMEM supplemented with 10% FBS and 1% antibiotics at 37 °C with 5% CO_2_. The culture medium was changed every 2 days.

### Preparation of CM

Third passage hADSCs and hFDSPCs were washed with PBS thrice and starved in DMEM for 48 h. Cell supernatants (500 mL) were collected, centrifuged at 300×*g* for 10 min, and filtered through a 0.22-μm filter to remove cell debris. The conditioned medium was then concentrated with a cut-off value of 10 kDa (Amicon Ultra-15, Millipore, MA, USA) and centrifuged at 3000×*g* for 1 h, eventually condensed into 25 mL. The concentrated CM was frozen and stored at − 80 °C until use. Before application, CM was quantified using a BCA Protein Assay Kit (Beyotime, Shanghai, China, Cat: P0012S) according to the instruction of the kit.

### Colony forming unit (CFU) assay

Passages 1–3 of hFDSPCs were cultured and plated in 6-well plates at a density of 50 cells/cm^2^. After 7 days, individual clones were identified under an inverted microscope (Leica, Wetzlar, Germany), fixed with 4% paraformaldehyde, and stained with 2% crystal violet for 15 min. Colonies containing 10 or more cells were selected, and the number of cells per colony was quantified. The experiment was repeated three times.

### Flow cytometry analysis of cell surface marker expression

The hFDSPCs or hADSCs (passage 3) cell suspension was prepared at a density of 10^6^ cells per 100 μL. Thereafter, antibodies (10 μL) were added to each 100 μL cell suspension, followed by incubation at room temperature for 30 min and analysis using flow cytometry (FACSCalibur, BD Biosciences, Mississauga, Canada).

All antibodies were purchased from BioLegend (San Diego, CA, USA) including the following: FITC-conjugated antibodies for CD90 (Cat: 328107), CD44 (Cat: 338803), CD105 (Cat: 323203), CD34 (Cat: 343603), CD45 (Cat: 368507), CD19 (Cat: 392507) or PE-conjugated antibodies CD29 (Cat: 303003), CD13 (Cat: 301703), CD59 (Cat: 304707), CD31 (Cat: 303105), CD133 (Cat: 372803), CD11b (Cat: 301305), HLA-DR (Cat: 307605), and CD73 (Cat: 344003). Isotype control IgG (Cat: 400107; 400111) was used to stain the cells as controls. The experiment was repeated three times.

### Induction of osteogenic, chondrogenic, and adipogenic differentiation

A trilineage-induced differentiation experiment including osteogenesis, adipogenesis, and chondrogenesis of hFDSPCs or hADSCs was performed to identify multiple differentiation potential. Briefly, hFDSPCs (passage 2) were cultured in 6-well plates at a density of 5 × 10^4^/cm^2^ with human MSC osteogenic and adipogenic differentiation medium (Cyagen Biosciences Inc., Sunnyvale, USA) for adipogenesis or osteogenesis induction, respectively. After a 2-week culture, alizarin red and oil red O staining was employed to evaluate osteogenesis and adipogenesis, respectively. For chondrogenesis, 3 × 10^5^ cells were resuspended in a 15-mL polypropylene centrifuge tube and centrifuged at 250×*g* for 4 min, and then resuspended in 0.5 mL human MSC chondrogenic differentiation medium (Cyagen Biosciences Inc., Sunnyvale, USA) and centrifuged at 150×*g* for 5 min. The pellet was cultured for 3 weeks, and then fixed in 4% paraformaldehyde and stained with Alcian blue.

### Animals

All experiments were approved and performed under the guidelines of the Ethics Committee of Shanghai Jiao Tong University School of Medicine. Male C57BLKS/J db/db diabetic mice (age, 8 weeks; weight, 36.5–40.2 g) were purchased from GemPharmatech Co., Ltd. (Jiangsu, China). Mice were fed and maintained under a 12-h light/dark cycle at an ambient temperature of 23–25 °C with 55–65% humidity. Mice were given standard rodent chow and water ad libitum.

### Diabetic mouse wound healing model

The mice were randomly divided into four groups (*n* = 5): (1) control group, treated with 100 μL PBS; (2) HA group, treated with 100 μL 1.5% HA (Sodium hyaluronate, Sigma-Aldrich, Missouri, USA, Cat: 63357, isolated from *Streptococcus equi* with a molecular weight of 1,500,000–1,750,000); (3) hADSC-CM + HA group, treated with 100 μg/mL condensed hADSC-CM in 1.5% HA (100 μL in total); and (4) hFDSPC-CM + HA group, treated with 100 μg/mL hFDSPC-CM in 1.5% HA (100 μL in total). All the mice were anaesthetised with isoflurane inhalation. After being shaved and depilated, a 6-mm-diameter circle, single, dorsal, full-thickness wound (including panniculus carnosus) was produced. The treatment was administered to all mice 1 day after the wound model was established as follows: sodium hyaluronate powder was dissolved in deionised water to form a 1.5% hydrogel (w/v). Condensed hFDSPC-CM/hADSC-CM (10 μg) was mixed with 100 μL 1.5% hydrogel and shaken by vortexing to form hADSC-CM + HA/ hFDSPC-CM + HA mixture for application. A total of 100 μL HA hydrogel or the above mixture was applied to the wound area. The hydrogel was very sticky, so no extra measure was taken to fix it, and this sticky feature enabled the CM to remain on the wound area.

### Gross evaluation of wound closure

Digital photographs of the wound were captured on days 0, 3, 7, 10, and 14. Time to wound closure was defined as the time at which the wound bed was completely re-epithelised. The wound areas were analysed by tracing the wound margins and calculated using Image-Pro Plus software version 6.0 (Media Cybernetics, Rockville, USA). The closure rate was expressed as a percentage area of the original wound area.

### Histological staining

On day 14, mice were sacrificed and full-thickness, cross-sectional tissue samples were obtained (specimens traversed the entire diameter of the wound and included unwounded skin on both sides). Specimens were then fixed in 4% paraformaldehyde, paraffin-embedded, and sectioned at 8 μm. Haematoxylin and eosin (HE) and Masson’s trichrome staining was performed for histological analyses.

For immunohistochemistry, samples from three animals (*n* = 3) in each group were sectioned and analysed for quantification of CD31, CD68, CD86, and CD206. Specimen sections were incubated with anti-CD31 (Abcam, Cambridge, UK, Cat: ab76533, 1:1000), anti-CD68 (Abcam, Cambridge, UK, Cat: ab213363, 1:2000), anti-CD86 (Abcam, Cambridge, UK, Cat: ab119857,1:200), and anti-CD206 (Abcam, Cambridge, UK, Cat:60143-1-lg, 1:1000) at 37 °C for 2 h, followed by incubation with horseradish peroxidase- (Jackson ImmunoResearch, Madison, USA, Cat: 111-035-003, 1:1000) or PE-conjugated secondary antibody (Jackson ImmunoResearch, Madison, USA, Cat:111585003, 1:1000). Haematoxylin or DAPI (1:1000, Boster, Wuhan, China, Cat: AR1176) was used to stain cell nucleus. The sections were examined under light microscopy (Leica, Wetzlar, Germany). Five randomly selected images of each section were used for quantification. CD31 quantification was performed by calculating CD31-positive tube number. CD68, CD86, and CD206 quantification was performed by calculating CD68-positive cell number.

For Picrosirius red staining, specimens from three animals (*n* = 3) were dewaxed in 100% xylene, followed by washing in 100% ethanol and PBS twice, and then were immersed in Picrosirius red (Sirius Red 0.1% in picric acid) for 1 h at room temperature. After washing in PBS, sections were rapidly dehydrated, cleared in xylene, and mounted. Collagen fibres were detected by light and polarised light microscopy (Olympus, Hamburg, Germany). Under polarised light microscopy, collagen I (COL 1) fibres were stained red, whereas collagen III (COL 3) fibres appeared green. Images were analysed using Image-Pro Plus 6 software (Rockville, MD, USA) as previously described [20]. To define the pixel count, ranges were selected in the red, green, and blue channels, and then through trial and error, we selected the colour green or red and calculated the area of each collagen type in one field. Five random fields were selected from each sample for statistical analysis.

### Cell viability assay

Briefly, 1000 cells (HaCaT and human fibroblasts) were suspended in 100 μL culture medium, seeded in 96-well plates, and cultured for 24 h. Thereafter, cells were separately treated with hADSC-CM and hFDSPC-CM at six concentrations: 0, 5, 10, 20, 50, and 100 μg/mL, followed by culture for 72 h. The cell viability assay was performed using cell counting kit-8 (CCK-8; Dojindo, Japan) according to the manufacturer’s instructions. Briefly, 10 μL CCK-8 solution was added to each well and incubated for 2.5 h at 37 °C. Next, the medium was harvested and measured at 450 nm using a microplate reader (Thermo Electron Corporation, Finland). All assays were repeated three times.

### EdU incorporation assay

HaCaT and human fibroblasts were seeded in 96-well plates (2000 cells per well) and incubated at 37 °C for 24 h. Cells were treated with or without hADSC-CM and hFDSPC-CM at a concentration of 20 μg/mL and continuously cultured for 72 h. Cells were treated with 5-ethynyl-20-deoxyuridine (BeyoClick™ EdU Cell Proliferation Kit with Alexa Fluor 488) at a working concentration of 50 μM in 100 μL culture medium for 2 h. Then, cells were washed twice with PBS for 10 min and fixed with 4% paraformaldehyde for 15 min at room temperature, followed by incubation with 0.5% Triton X-100 (Sigma). Cells were then counterstained with DAPI (1:1000, Boster, Wuhan, China) and imaged under a fluorescent microscope (Olympus, Tokyo, Japan). Five randomly selected fields from each well were imaged, and the EdU-positive cells were calculated using Image-Pro Plus 6 software (Rockville, MD, USA).

### Scratch assay

Migration of HaCaT and human fibroblasts was measured using the monolayer wound assay in vitro. Cells were plated in 6-well plates (1 × 10^5^ cells per well) with culture medium until completely confluent. Cells were scraped across the plate with a 200-μL pipette tip. Cells were cultured with serum-free DMEM with or without hADSC-CM and hFDSPC-CM (20 μg/mL) for 24 h. Cell migrations at 0 and 24 h were imaged with inverted microscopy (Olympus, Tokyo, Japan), and five randomly selected fields from each well were used for scratch area calculation using Image-Pro Plus 6 software. The results are presented as scratch area at 0 h − scratch area at 24 h (μm^2^).

### Real-time-qPCR (RT-qPCR)

Total RNA was extracted from cells using TRIzol® reagent (Invitrogen, Carlsbad, USA), followed by treatment with DNase I (Promega Corp., Madison, USA). cDNA was synthesised using a high-capacity cDNA synthesis kit (Takara Bio, Inc., Tokyo, Japan) according to the manufacturer’s instructions. RT-qPCR was performed to determine mRNA levels using SYBR-Green I (Takara Bio, Inc. Otsu, Japan). The thermal cycling parameters were 95 °C for 1 min, followed by 40 cycles at 95 °C for 10 s, and 60 °C for 40 s. The expression levels of genes were normalised to *β-actin* housekeeping gene expression. The primers used for real-time qPCR analysis are listed in Table 1.

**Table 1:** Primer sequences used for real-time qPCR

Gene	Species	Forward primer	Reverse primer
ALP	Human	TACAAGCACTCCCACTTCATC	AGACCCAATAGGTAGTCCACAT
BMP-2	Human	GAAGAACTACCAGAAACGAGTG	GGTGATGGAAACTGCTATTG
Osx	Human	CAGTTGATAGGGTTTCTCTTGTA	CATAGGACTTGAGGTTTCACAG
OCN	Human	CTGTGACGAGTTGGCTGAC	AGCAGAGCGACACCCTAGA
OPN	Human	CATTCCGATGTGATTGATAGTC	CTTCCTTACTTTTGGGGTCTAC
C/EBP α	Human	CCCTCAGCCTTGTTTGTACT	AAAATGGTGGTTTAGCAGAGA
FABP4	Human	AGAGAAAACGAGAGGATGATAA	TTCAATGCGAACTTCAGTC
PPAR-γ2	Human	GCAGTGGGGATGTCTCATAAT	CAGCGGACTCTGGATTCAG
SREBP1	Human	AACACAGCAACCAGAAACTCA	GTCCTCCACCTCAGTCTTCAC
COL 2	Human	TGGACGCCATGAAGGTTTTCT	TGGGAGCCAGATTGTCATCTC
Aggrecan	Human	GTGCCTATCAGGACAAGGTCT	GATGCCTTTCACCACGACTTC
SOX-9	Human	AGCGAACGCACATCAAGAC	CTGTAGGCGATCTGTTGGGG
COL 1	Human	AGGGCCAAGACGAAGACATC	GTCGGTGGGTGACTCTGAGC
COL3	Human	AAGGGCAGGGAACAACT	ATGAAGCAGAGCGAGAAG
MMP-1	Human	GGAGCTGTAGATGTCCTTGGGGT	GCCACAACTGCCAAATGGGCTT
MMP-3	Human	AGGACAAAGCAGGATCACAGTTG	CCTGGTACCCACGGAACCT
FN	Human	TCTCCTGCCTGGTACAGAATAT	GGTCGCAGCAACAACTTCCAGGT
α-SMA	Human	GCTACTCCTTCGTGACCACAG	GCCGTCGCCATCTCGTTCT
TGF-β1	Human	TACTACGCCAAGGAGGTCAC	GAGAGCAACACGGGTTCAG
β-actin	Human	GGCACTCTTCCAGCCTTCC	GAGCCGCCGATCCACAC

### Western blot analysis

Human fibroblasts were seeded into 6-well plates (2 × 10^5^ cells/ml) and cultured with or without hADSC-CM and hFDSPC-CM at a concentration of 20 μg/mL for 48 h. After removal of the medium, cells were washed with PBS twice, and then lysed using RIPA Lysis Buffer (Beyotime, Shanghai, China) with 1 mM phenylmethanesulfonyl fluoride (PMSF, Beyotime, Shanghai, China). The lysates were collected and centrifuged at 14,000×*g* at 4 °C for 5 min. The supernatant protein concentration was quantified using a BCA Protein Assay Kit (Beyotime, Shanghai, China, Cat: P0012S) according to the instructions. The prepared protein was subjected to SDS-PAGE and subsequently transferred onto PVDF membranes. The PVDF membrane was blocked with 5% non-fat powdered milk in Tris-buffered solution plus Tween-20 (TBST) for 2 h at 37 °C. Membranes were then incubated overnight at 4 °C with primary antibodies, followed by incubation with appropriate HRP-conjugated secondary antibodies (Jackson ImmunoResearch, Madison, USA, Cat:111-035-003). The protein bands were visualised using an enhanced chemiluminescence (ECL) detection kit (Amersham Pharmacia Biotech, Piscataway, USA). The primary antibodies, including COL1 (Cat: ab138492), COL3 (Cat: ab184993), fibronectin (FN, Cat: ab2413), α-SMA (Cat: ab124964), and transforming growth factor-beta 1 (TGF-β1, Cat: ab215715), were purchased from Abcam (Cambridge, UK). Primary antibodies, including phospho-Smad2 (Cat: 18338), phospho-Smad3 (Cat: 9520), and Smad2/3 (Cat: 5678 s) were purchased from Cell Signalling Technology (CST, NY, USA). The primary antibody for β-actin was purchased from Sigma-Aldrich (Missouri, USA, Cat: ZRB1312).

### Immunofluorescence staining

Human fibroblasts were seeded (1 × 10^5^ cells/mL) and cultured with or without hADSC-CM and hFDSPC-CM (20 μg/mL) for 48 h. Cells were then washed thrice with PBS and fixed with 4% paraformaldehyde for 15 min at room temperature. After nonspecific antigen blocking using goat serum (Beyotime, Shanghai, China), cells were incubated with COL 1(Cat:ab138492), COL 3 (Cat: ab237238), FN (Cat: ab45688), and α-SMA (Cat: ab124964) primary antibodies (1:250–1:1000, Abcam, Cambridge, UK) at 4 °C overnight, followed by incubation with secondary antibodies (1:150; Santa Cruz Biotechnology, Inc., CA, USA, Cat: sc-516248) for 1 h at 37 °C. Images were captured using an inverted fluorescence microscope, and five randomly selected pictures were used to calculate positive cell numbers.

### ELISA assay

After 72 h of incubation with or without hADSC-CM and hFDSPC-CM (20 μg/mL) in serum-free culture medium, TGF-β1 protein from human fibroblasts was measured using a Human TGF-β1 ELISA kit (ExCell Bio. Shanghai, China) according to the manufacturer’s instructions. All assays were performed three times.

### Tube formation assay

HUVECs (1 × 10^4^/well) were suspended in DMEM supplemented with 10% FBS, and then seeded onto Matrigel-coated 96-well plates with or without hADSC-CM and hFDSPC-CM (20 μg/mL). The plate was incubated at 37 °C in 5% CO_2_ for 6 h. Tube formation was photographed under a light microscope (Carl Zeiss, Oberkochen, Germany). The number of junctions was calculated using ImageJ software (NIH, Bethesda, MD, USA).

### Statistical analysis

Statistical analyses were performed using GraphPad Prism6 software (La Jolla, USA). Student’s unpaired *t* test was performed for two-group comparisons; one-way analysis of variance (ANOVA) was used for multiple group comparisons. All values in this study are presented as mean ± standard deviation. A probability (*p*) value < 0.05 was considered significant.

## Results

### Identification of hFDSPCs and hADSCs

**Fig. 1:**
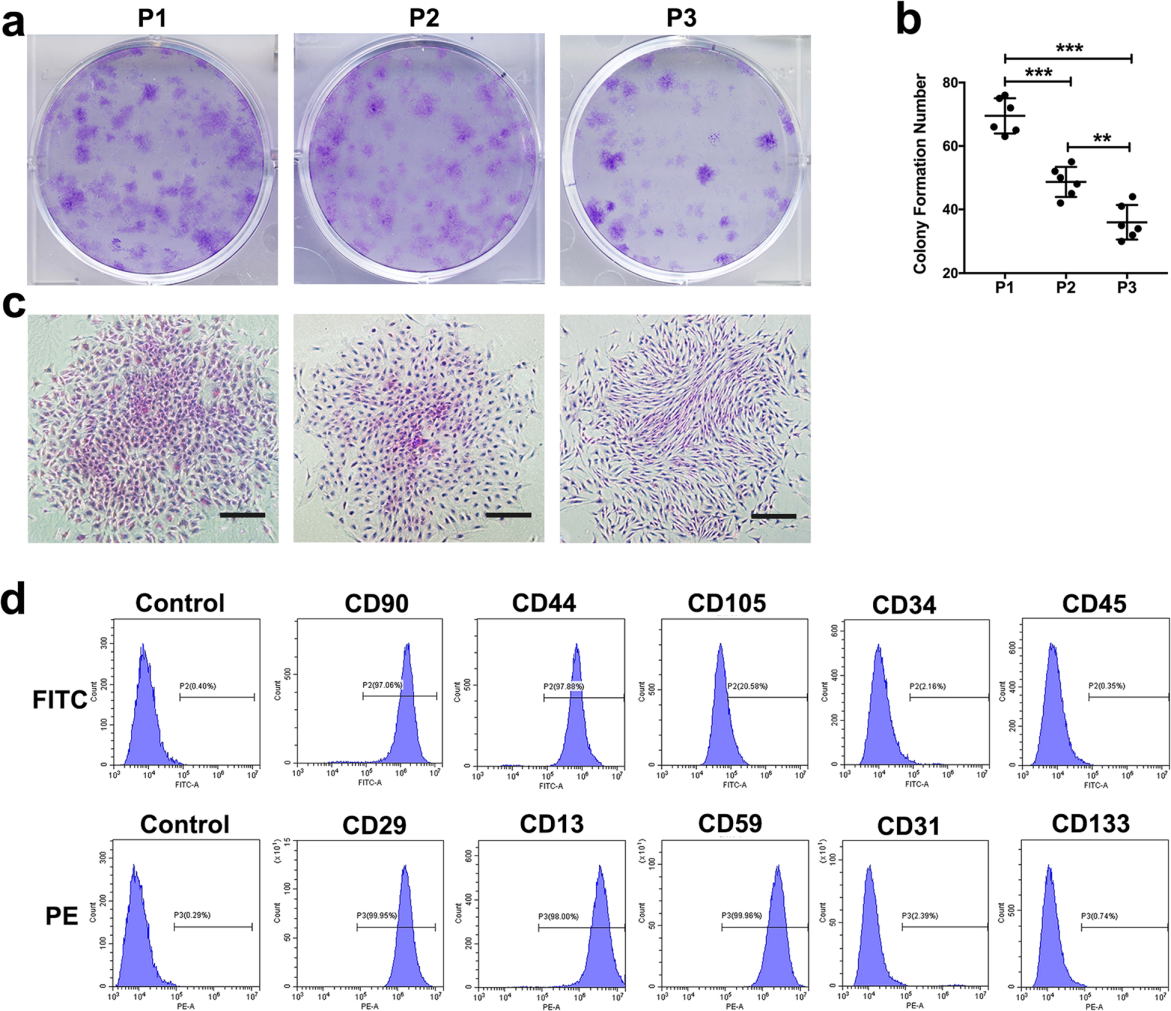
The clonogenic capacity and surface marker expression of hFDSPCs. The clonogenic capacity of hFDSPCs was examined using CFU assays. **a** hFDSPCs cultured at P1 to P3 were seeded in 6-well plates at a density of 50 cells/cm^2^, with cell clones observed after 7 days. **b** Colony formation numbers were counted; the data were plotted in graphs and analysed using Graph Pad Prism Software (*n* = 6). **c** The colony morphology was observed under an inverted microscope, bar = 1000 μm. **d** Immunophenotyping of hFDSPCs was characterised using flow cytometry analysis. hFDSPCs were analysed for expression of the following markers (*n* = 4): CD90 (97.53% ± 0.48%), CD44 (97.28% ± 0.52%), CD105 (22.57% ± 2.09%), CD34 (1.30% ± 0.75%), CD45 (0.73% ± 0.39%), CD29 (98.62% ± 1.18%), CD13 (98.06% ±0.26%), CD59 (98.80% ± 1.02%), CD31(2.07% ± 0.31%), and CD133 (0.88% ± 0.16%). Isotype-matching IgG-FITC and IgG-PE were used to determine nonspecific signals. Data are shown as means ± SD, **p < 0.01, ***p < 0.001

Passages 1–3 of hFDSPCs were cultured and formed compact and circular colonies. With increased cell passage (Fig. 1a), cell morphology gradually appeared more spindle-like (Fig. 1c), and colony formation number decreased, but remained above 30 (P1 = 69.50 ± 5.54, P2 = 48.67 ± 4.72, P3 = 36 ± 5.40, *n* = 6) (Fig. 1b). Flow cytometry results showed that hFDSPCs positively expressed CD90 (97.53% ± 0.48%), CD44 (97.28% ± 0.52%), CD105 (22.57% ± 2.09%), CD29 (98.62% ± 1.18%), CD13 (98.06% ±0.26%), and CD59 (98.80% ± 1.02%), but negatively expressed CD34 (1.30% ± 0.75%), CD45 (0.73% ± 0.39%), CD31(2.07% ± 0.31%), and CD133 (0.88% ± 0.16%) (Fig. 1d).

**Fig. 2:**
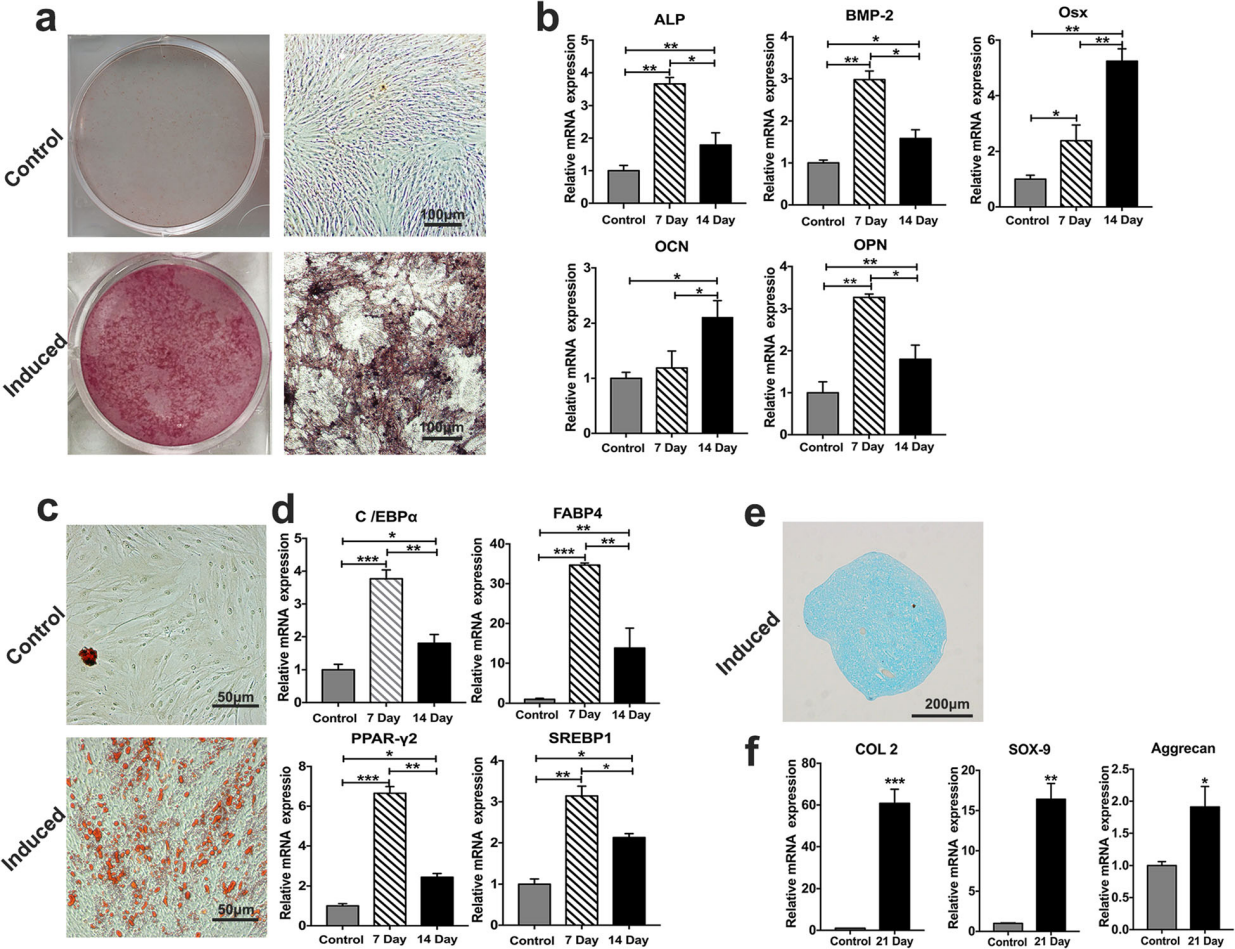
Multiple differentiation potential of hFDSPCs was examined using trilineage-induced differentiation experiment assays. Cells at passage 2 were used in all experiments. **a** The osteogenesis potential was examined using alizarin red staining, bar = 100 μm, **b** and the related gene expressions were analysed using RT-qPCR assays. **c** Adipogenesis was analysed using oil red O staining, bar = 50 μm, **d** and the related gene expressions were examined. Chondrogenesis was assessed using Alcian blue staining, bar = 200 μm (**e**), and RT-qPCR assays (**f**). Data are shown as means ± SD, *p < 0.05, **p < 0.01, ***p < 0.001

Passage 2 hFDSPCs showed osteogenic (Fig. 2a, b), adipogenic (Fig. 2c, d), and chondrogenic (Fig. 2e, f) differentiation potential. The elevated osteogenic (ALP, BMP-2, Osx, OCN, OPN), adipogenic (C/EBPα, FABP4, PPAR-γ2, SREBP1), and chondrogenic (COL2, Aggrecan, SOX-9) mRNA expression confirmed the trilineage differentiation results. In addition, in the chondrogenic differentiation assay, no pellet was formed in the control group during the culture process.

Passage 3 hADSCs showed high-level expression of hADSC surface markers, such as CD73 (97.97% ± 1.19%), CD90 (97.03% ± 1.45%), and CD105 (97.04% ± 1.12%), but almost no expression of negative markers, such as CD19 (1.17% ± 0.69%), CD34 (0.83% ± 0.35%), CD11b (1.50% ± 0.86%), CD45 (0.99% ± 0.54%), and HLA-DR (1.20% ± 0.45%) (Fig. S1).

### hFDSPC-CM promoted wound healing in db/db mice

**Fig. 3:**
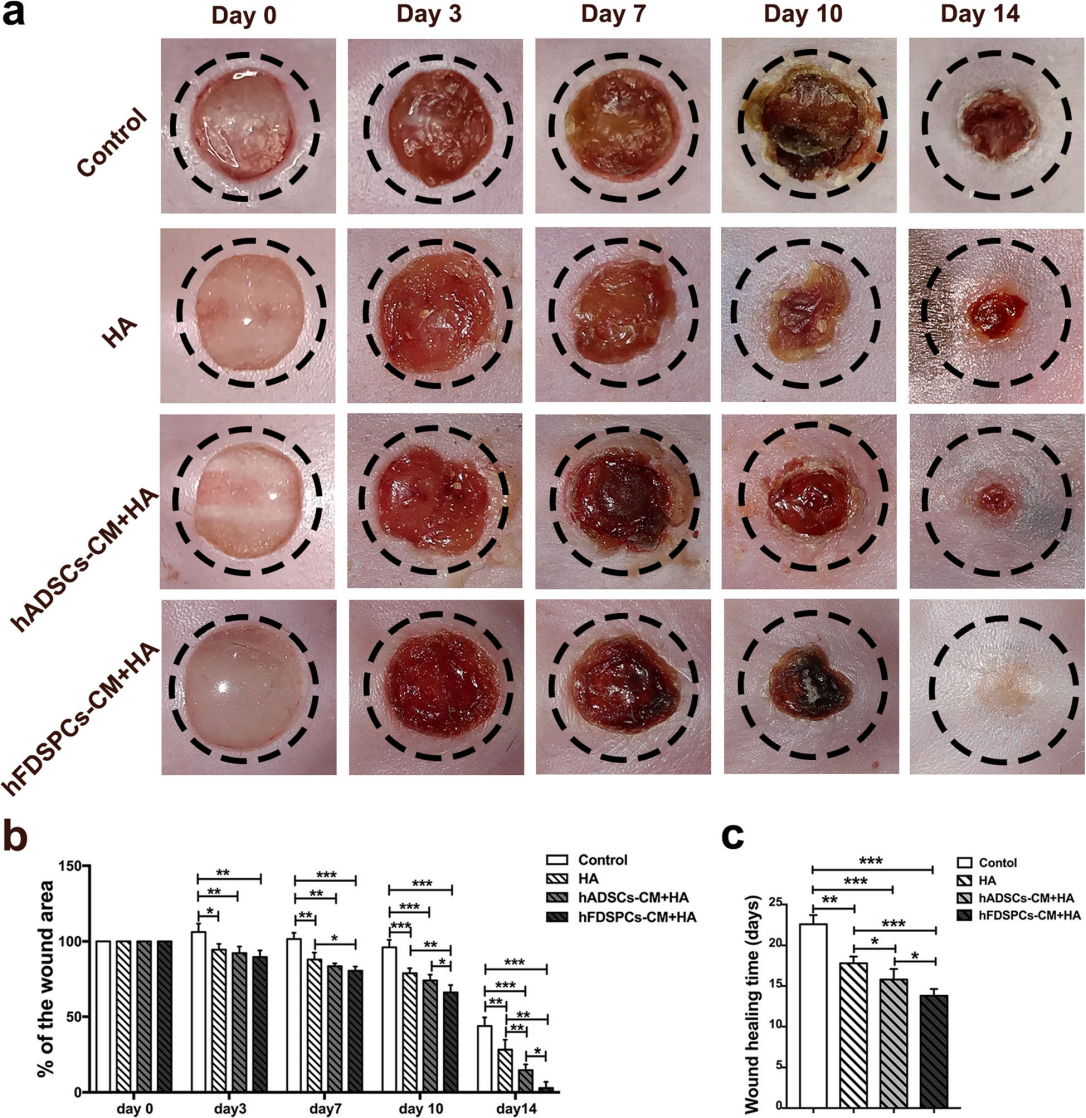
The effect of hFDSPCs on wound closure of full-thickness excisional wounds in db/db mice. Representative images of macroscopic view of db/db mice wound healing treated with 100 μL PBS (Control group), 100 μL 10% HA (HA group), 100 μg/mL hADSC-CM in 10% HA (hADSC-CM + HA group), and 100 μg/mL hFDSPC-CM in 10% HA hFDSPC-CM (hFDSPC-CM + HA group) on day 0 (before treatment), 3, 7, 10, and 14 post-wounding (**a**). **b** Percentage of wound area in each group at day 0, 3, 7, 10, and 14 post-wounding (*n* = 5/group). **c** The wound healing time of each group (*n* = 5/group). Data are shown as means ± SD; *p < 0.05, **p < 0.01, ***p < 0.001

During the wound healing process, the wounds in all groups remained clean and dry with few exudates, blood scabs, and no obvious contraction. The HA hydrogel or hydrogel mixture was observed to be gradually absorbed within 48 h. Regarding the wound area, HA treatment, hADSC-CM + HA, and hFDSPC-CM + HA groups showed significantly decreased wound area compared to the control group from day 3 to day 14 (*p* < 0.05). Furthermore, the hFDSPC-CM + HA group showed significantly decreased wound area compared to the HA group from day 7 and significantly decreased the wound area compared to the hADSC-CM group from day 10 (Fig. 3a, b) (*p* < 0.05). The results demonstrated that HA combined with hFDSPC-CM exhibited the best curative effect in promoting wound healing over time.

Regarding wound healing time, all treatment groups showed significantly accelerated wound healing compared to the control group (*p* < 0.05). Further, the HA + hFDSPC-CM group exhibited the shortest wound healing time (13.80 ± 0.84 days) compared to the control group (22.6 ± 1.14 days), as well as the HA group (17.80 ± 0.84 days) and HA + hADSC-CM group (15.8 ± 1.30 days) (Fig. 3c).

### Assessment of the effect of hFDSPC-CM on re-epithelialisation, angiogenesis, and anti-inflammation in db/db mice wound

**Fig. 4:**
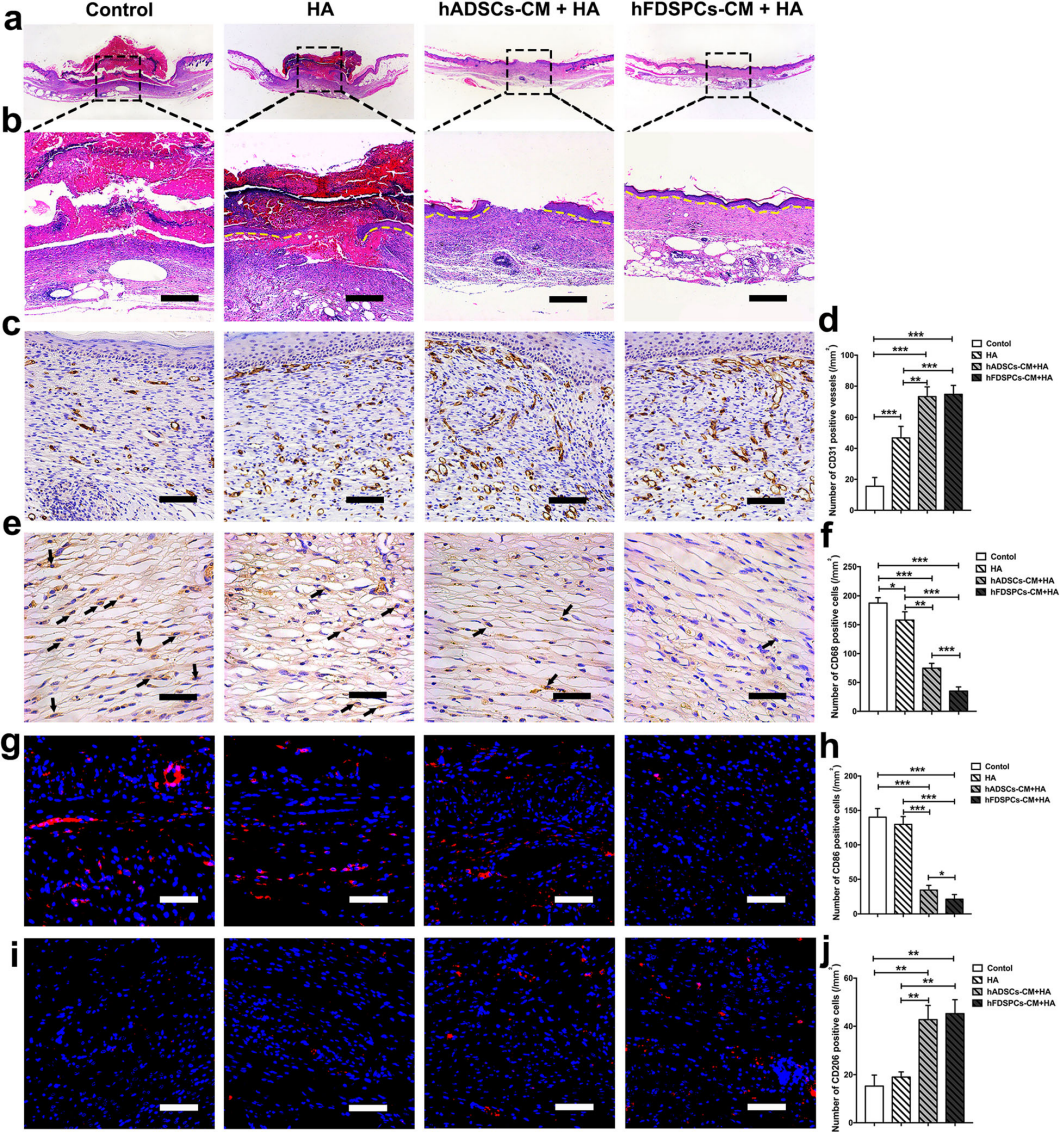
The effect of hFDSPCs on inflammation in wound of db/db mice was evaluated via optical microscopy analysis. **a** Re-epithelialisation on day 14 was analysed using H&E staining in wound tissue sections, and photos were taken with a × 1.25 lens. **b** Re-epithelialisation is indicated by the yellow dotted line, bar = 400 μm. **c** CD31-stained wound tissues on day 14 were shown via IHC assay, bar = 200 μm. **d** Statistical analysis of number of CD31-positive vessels in the wound tissues. **e** CD68 staining results showed infiltration of macrophages/monocytes on day 14, and the black arrows indicate accumulation of CD68-positive cells in wound tissues, bar = 100 μm. **f** The number of CD68-positive cells on day 14 was counted. CD86 (**g**) (red) and CD206 (**i**) (red) staining results showed M1 or M2 type macrophage polarisation on day 14. The number of CD86- (**h**) and CD206- (**j**) positive cells on day 14 was counted, bar = 100 μm. Data are shown as means ± SD; *n* = 5; *p < 0.05, **p < 0.01, ***p < 0.001

Granulation tissue formation and re-epithelialisation on day 14 was visualised using HE staining (Fig. 4a, b). Histological observations showed that there was almost no epidermis in the control group, while little re-epithelialisation could be observed at the edge of the wound in the HA group. The newly formed tissue in the hADSC-CM + HA group was almost intact, but the epidermal tissue remained discontinuous. In contrast, the wound tissue in the hFDSPC-CM + HA group already showed intact and thinner epithelium in comparison to other groups.

Wound angiogenesis was assessed using CD31 staining (Fig. 4c). Compared to the control group, all other groups showed increased microvessels in the granulation tissue at day 14 (*p < 0.001*). Besides, both hFDSPC-CM + HA group and hADSC-CM + HA group showed increased microvessel density compared to the HA group (*p < 0.01*). However, no significant difference was observed between the hADSC-CM + HA and hFDSPC-CM + HA groups (Fig. 4d). These results indicate that hFDSPC-CM exerts a similar effect on wound angiogenesis as hADSC-CM in diabetic mice.

Macrophages stained for CD68 (general macrophages), CD86 (M1 type macrophages), and CD206 (M2 type macrophages) were used to evaluate inflammatory cell infiltration and macrophage polarisation (Fig. 4e, g, i). Compared to the control group, all treatment groups showed significantly decreased CD68-positive cells (*p < 0.05*). In addition, both the hFDSPC-CM + HA and hADSC-CM + HA groups showed significantly decreased CD68-positive cells compared to the HA group (*p < 0.01*). Furthermore, the hFDSPC-CM + HA group showed the least CD68-positive cells compared with the other groups (Fig. 4f). In the hFDSPC-CM + HA and hADSC-CM + HA groups, CD86-positive cells significantly decreased (*p < 0.001*), while CD206-positive cells significantly increased (*p < 0.01*) compared to those in the HA and control group. The HA group showed no significant difference in CD86- and CD-206 positive cells compared to the control group (*p* > 0.05). However, the hFDSPC-CM+ HA group showed less CD86-positive cells (*p <* 0.05), but comparable CD206-positive cells (*p* > 0.05) when compared with the hADSC-CM + HA group (Fig. 4h, j). These results indicate that hFDSPC-CM inhibited excessive inflammation and increased anti-inflammation response in diabetic wound healing processes.

### hFDSPC-CM enhanced collagen regeneration, maturation, and remodelling in db/db mouse wounds

**Fig. 5:**
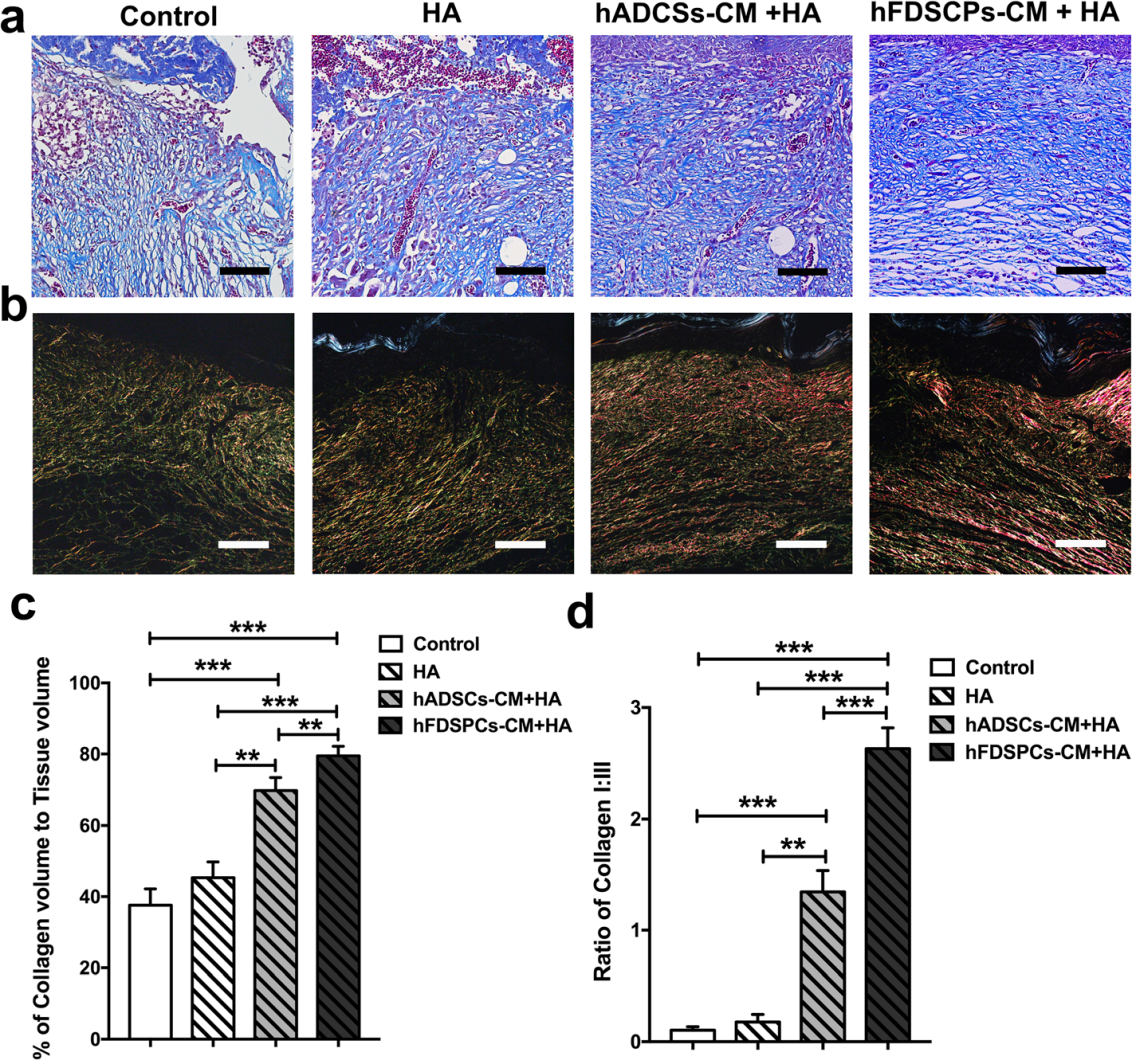
Effect of hFDSPC-CM on collagen regeneration, maturation, and remodelling in wounds of db/db mice. **a** Masson staining for representative wound beds on day 14; collagen deposition is stained blue, bar = 200 μm. **b** Regenerated collagen content was analysed using Picrosirius red staining under a polarised light microscope. Collagen type III is visualised in green colour and collagen type I is visualised in orange/red colour, bar = 400 μm. **c** The percentage of collagen volume to tissue volume was quantified according to the Masson staining results. **d** Ratio of collagen I:III in wound beds of each group. Data are shown as means ± SD; *n* = 4; **p < 0.01, ***p < 0.001

The regeneration of dermis in diabetic wounds is mainly assessed by collagen regeneration and remodelling through Masson’s trichrome staining and Picrosirius red staining. As shown in Fig. 5a, the Masson’s trichrome staining results were similar to HE staining results in each group on day 14. Newly formed sparse collagen tissue components were found in the control group. In the HA group, collagen formation increased but the dermis layer remained incomplete. By contrast, both the hFDSPC-CM + HA and hADSC-CM + HA groups showed more collagen synthesis than the HA and control groups (*p < 0.01*) (Fig. 5b). Further, the hFDSPC-CM + HA group exhibited the greatest collagen synthesis and most orderly collagen arrangement (Fig. 5c).

As the results showed, in the control and HA group, collagen I/III ratio was less than 0.5 (collagen I/II = 0.13 ± 0.03 and 0.18 ± 0.07, respectively in the control and HA groups), with no significant difference between the two groups (*p > 0.05*). However, both the hFDSPC-CM + HA and hFDSPC-CM + HA groups showed significantly increased collagen I/III ratio (collagen I/II = 1.35 ± 0.19 and 2.63 ± 0.19, respectively) (*p < 0.001*). Furthermore, the hFDSPC-CM + HA group exhibited a higher ratio than the hADSC-CM + HA group, indicating that hFDSPC-CM mainly promoted collagen I expression rather than collagen III expression (Fig. 5b, d).

### hFDSPC-CM promoted cell proliferation of HaCaT cells and human fibroblasts in vitro

**Fig. 6:**
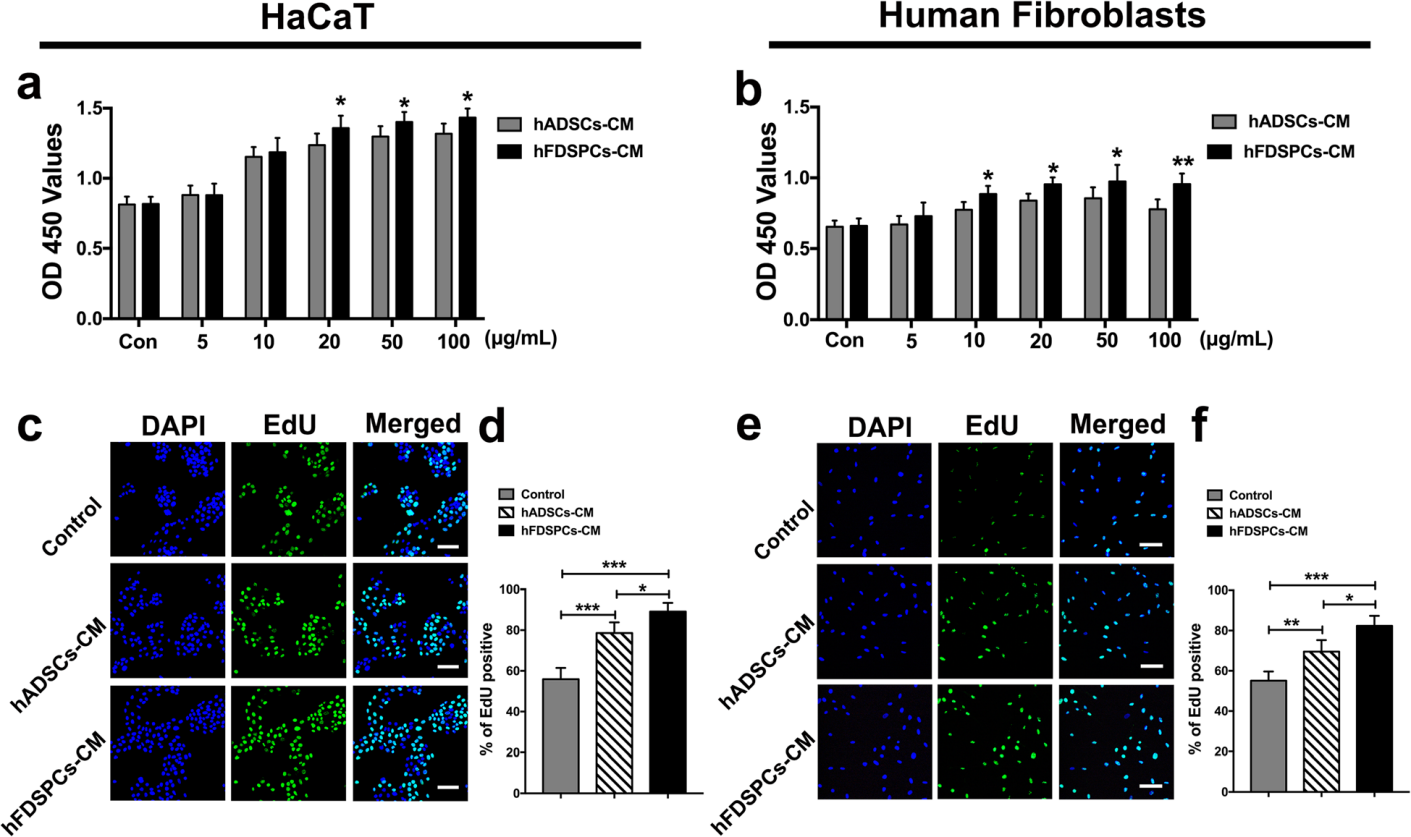
Effect of hFDSPC-CM on cell proliferation of HaCat and human fibroblasts in vitro. HaCat and human fibroblasts were treated with hFDSPC-CM or hADSC-CM at different concentrations (0, 5, 10, 20, 50, 100 μg/mL) for 72 h. Then, the CCK-8 assay was used to test the effects on HaCat (**a**) and human fibroblast (**b**) proliferation. Cell proliferative activity was assessed using the EdU incorporation assay. The cells were treated with PBS/hFDSPC-CM/hADSC-CM at a concentration of 20 μg/mL and separately and continuously cultured for 72 h. EdU-positive stained HaCat are shown (**c**), and the percentage of EdU-positive cells was measured from four randomly selected fields (**d**). EdU-positive stained human fibroblasts are shown (**e**), and the percentage of EdU-positive cells was measured from four randomly selected fields (**f**). Data are shown as means ± SD; bar = 100 μm; *n* = 4, *p < 0.05, **p < 0.01, ***p < 0.001

To explain the mechanism of hFDSPC-CM in promoting wound re-epithelialisation and collagen synthesis, which were mainly attributed to epidermal cells and dermis cell biological activity, HaCaT cells and human fibroblasts were treated with various concentrations of hFDSPC-CM or hADSC-CM (0, 5, 10, 20, 50, 100 μg/mL) for 72 h. CCK-8 cell activity (metabolic activity) assay results showed that both hFDSPC-CM and hADSC-CM promoted HaCaT cell/human fibroblast activity in a dose-dependent manner (Fig. 6a, b). For HaCaT cells, hFDSPC-CM exhibited an improved effect in promoting cell activity compared to hADSC-CM at 20 μg/mL and 100 μg/mL (*p < 0.05*). For human fibroblasts, hFDSPC-CM promoted cell activity compared to hADSC-CM in all treatment groups (*p < 0.05*). Based on the above results, 20 μg/mL was chosen as an effective concentration to perform the following in vitro experiments. EdU assay results indicated that HaCaT cell/human fibroblasts significantly proliferated compared to the control group (*p < 0.01*) after 72 h of incubation with hFDSPC-CM and hADSC-CM, and hFDSPC-CM exerted stronger effects on the proliferation of both HaCaT cells and human fibroblasts (*p < 0.05*).

### HFDSPC-CM promoted cell migration of HaCaT and human fibroblasts in vitro

**Fig. 7:**
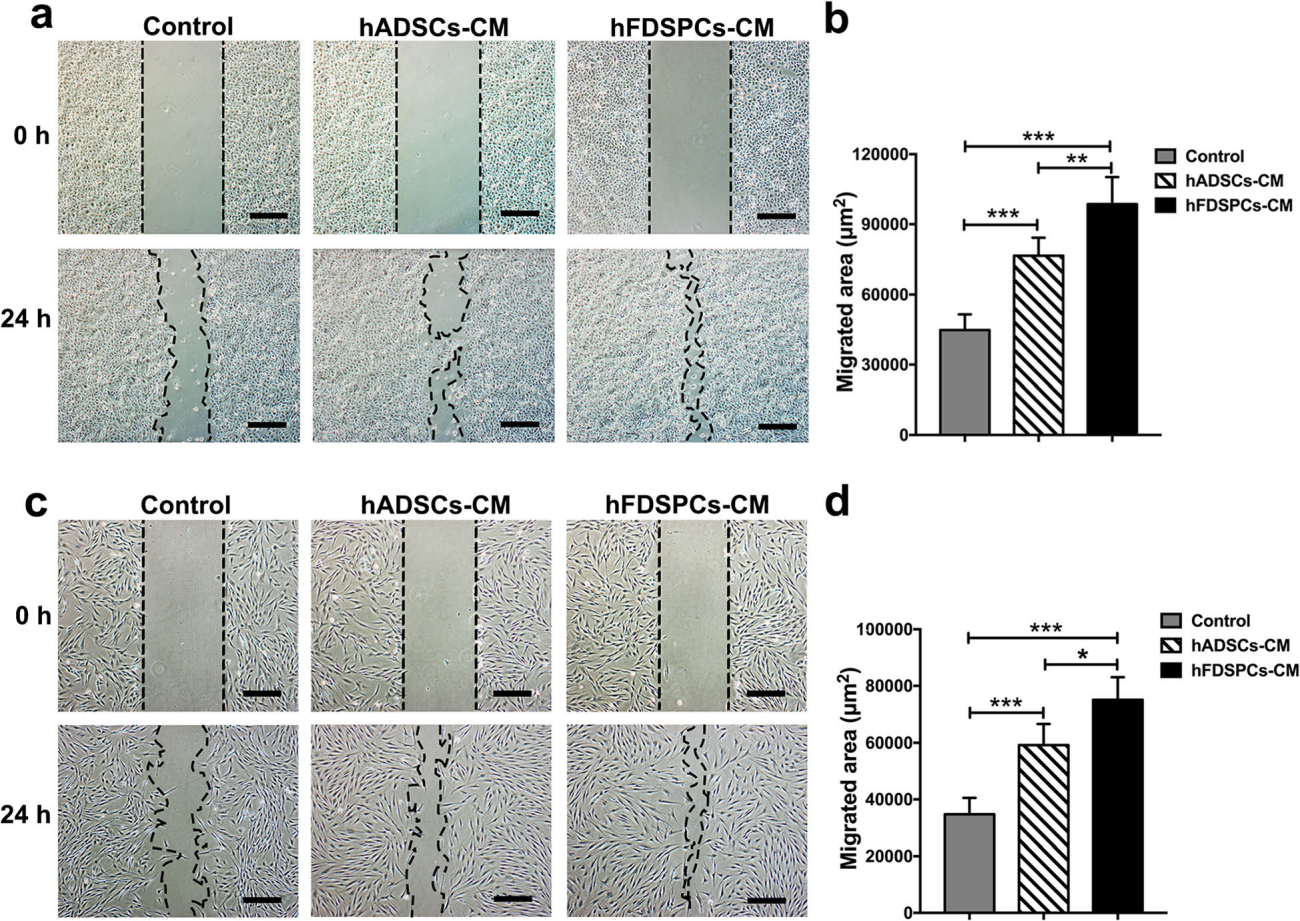
Effect of hFDSPC-CM on cell migration of HaCat and human fibroblasts was tested using the scratch assay in vitro. The scratch was made when cultured cells reached a confluent monolayer. Then, cells were treated with PBS/hFDSPC-CM/hADSC-CM at a concentration of 20 μg/mL and cultured for 24 h. The data acquired from four randomly selected fields were quantified as a percentage of the area of the scratch filled with cells. HaCaT migration was observed at 0 and 24 h (**a**), and the migrated area was measured (**b**). Human fibroblast migration was evaluated (**c**), and the migrated area was quantified (**d**). Data are shown as means ± SD; bar = 100 μm; *n* = 4; *p < 0.05, ***p < 0.001

After incubation with 20 μg/mL hFDSPC-CM/hADSC-CM for 24 h, the migration area of HaCaT cells and human fibroblasts significantly increased in the hFDSPC-CM and hADSC-CM group, compared to that in the control group (Fig. 7). Regarding human fibroblasts, the hFDSPC-CM group demonstrated a larger migration area than the hADSC-CM group (*p < 0.05*) (Fig. 7d). However, no significant difference was observed in HaCaT cell migration between the hFDSPC-CM and hADSC-CM groups (*p > 0.05*) (Fig. 7b).

### HFDSPC-CM promotes tube formation of HUVECs in vitro

In vitro, hFDSPC-CM and hADSC-CM significantly promoted tube formation compared to the control group. However, no significant difference was observed between the hFDSPC-CM and hADSC-CM groups (*p > 0.05*), which indicates that hFDSPC-CM and hADSC-CM display equal efficiency in promoting tube formation of HUVECs (Fig. S2).

### HFDSPC-CM promotes extracellular matrix (ECM) production of human fibroblasts by activating TGF-β/Smad pathways in vitro

**Fig. 8:**
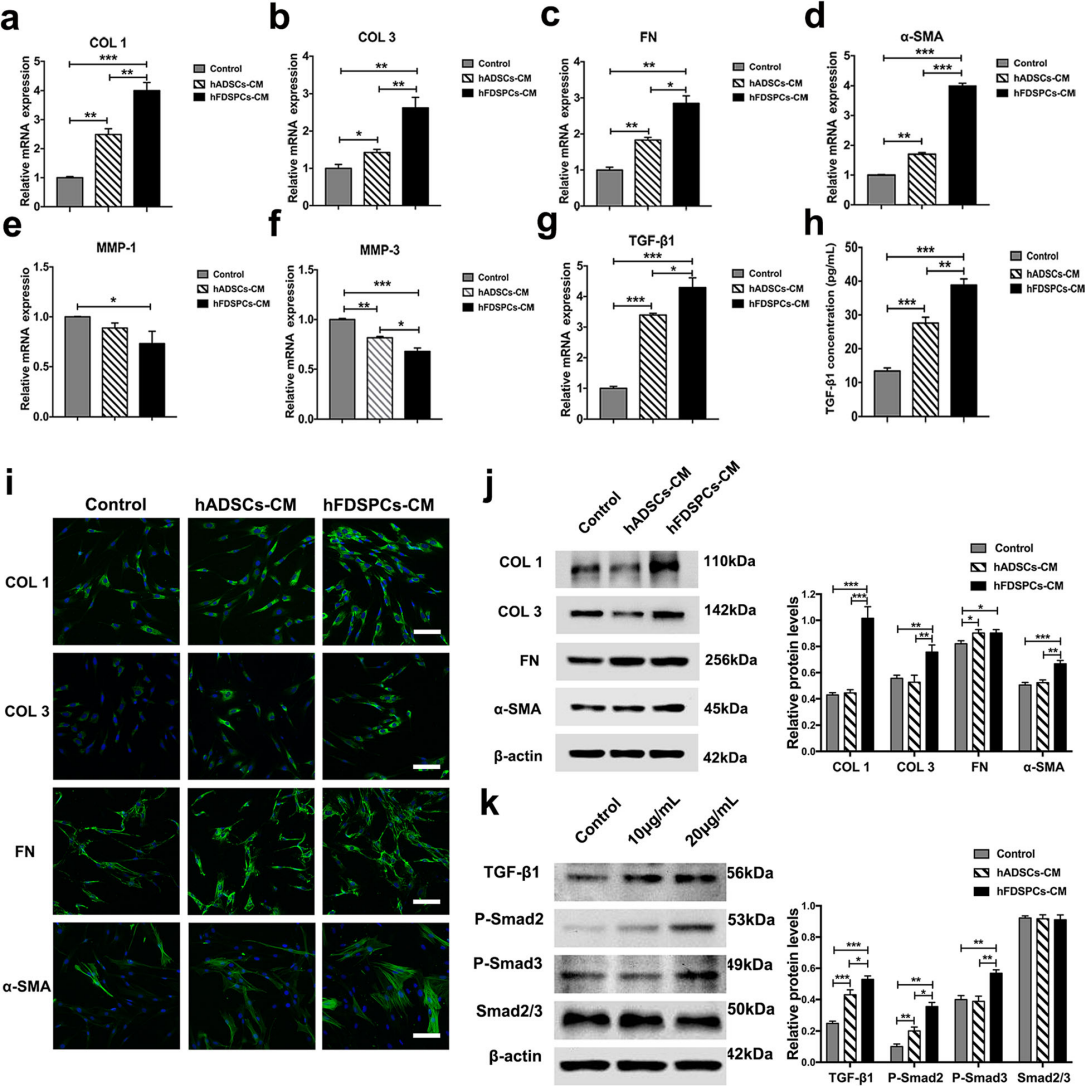
hFDSPC-CM promotes collagen production of human fibroblasts through the TGF-β/Smad signalling pathway in vitro. After treatment with hFDSPC-CM or hADSC-CM (20 μg/mL) for 48 h, the role on gene expression levels of COL 1 (**a**), COL 3 (**b**), FN (**c**), α-SMA (**d**), MMP-1 (**e**), MMP-1 (**f**), and TGF-β1 (**g**) was assessed using RT-qPCR in human fibroblasts. **h** After incubation with hADSC-CM or hFDSPC-CM (20 μg/mL) in serum-free culture medium for 72 h, TGF-β1 protein from human fibroblasts was measured using an ELISA assay. **i** ECM components, COL 1, COL 3, and FN, and activated fibroblast markers, α-SMA, were assessed using immunofluorescence analysis in human fibroblasts incubated with hADSC-CM or hFDSPC-CM (20 μg/mL), bar = 50 μm. **j** Human fibroblasts were treated with increasing doses of hFDSPC-CM (0, 10, and 20 μg/mL) for 48 h and harvested for western blot analysis to assess the intracellular signalling as indicated. **k** Human fibroblasts were analysed for activation of the TGF-β/Smad pathway after 48 h incubation with PBS/10 μg/mL or 20 μg/mL hFDSPC-CM. Data are shown as means ± SD; bar = 100 μm; *n* = 4; *p < 0.05, *p < 0.01, ***p < 0.001

We observed that hFDSPC-CM enhanced wound collagen synthesis, maturation, and remodelling, the mechanism of which may be attributed to fibroblast functions. Thus, human fibroblasts were incubated with hFDSPC-CM for 48 h and the functions related to the dynamic balance of extracellular matrix metabolism were analysed. mRNA and protein expression results showed that hADSC-CM and hFDSPC-CM significantly promoted ECM marker production, such as COL1, COL3, and FN. hFDSPC-CM demonstrated improved promotion of markers compared with hADSC-CM (Fig. 8a, b, c, i, j). Meanwhile, compared to the control group, both hADSC-CM and hFDSPC-CM significantly decreased matrix metalloproteinases 3 (MMP-3), which plays a role in ECM degradation (*p < 0.01*). Significantly decreased MMP-3 expression was also observed in the hFDSPC-CM group compared to the hADSC-CM group. hFDSPC-CM rather than hADSC-CM significantly decreased matrix metalloproteinases 1 (MMP-1) expression compared to the control group (*p < 0.05*).

α-SMA as a marker of myofibroblasts, which contract the wound to accelerate wound healing, was also detected. mRNA and protein expression results showed that hFDSPC-CM and hADSC-CM significantly increased α-SMA expression. Meanwhile, greater α-SMA expression was observed in the hFDSPC-CM group than in the hADSC-CM group.

The TGF-β1/Smad signalling pathway is pivotal for fibroblast differentiation and ECM deposition in wound healing [21]. Hence, based on the above results, we further investigated whether TGF-β1/Smad signalling in human fibroblasts was unregulated under hFDSPC-CM exposure. Firstly, hFDSPC-CM increased TGF-β1 transcript and extracellular secretion in comparison with other groups (Fig. 8g, h). Moreover, western blot analysis showed that hFDSPC-CM increased the expression of TGF-β1 and phosphorylation levels of Smad 2 and Smad 3 in a dose-dependent manner (Fig. 8k). These observations suggested that both TGF-β/Smad pathways were activated in human fibroblasts exposed to hFDSPC-CM.

Taken together, these data revealed that hFDSPCs can promote collagen production of human fibroblasts through TGF-β/Smad pathways.

## Discussion

As a biological waste, human foreskin is a reservoir of abundant dermal stem/progenitor cells with potential therapeutic value. However, no research has investigated the use of hFDSPC-CM to facilitate diabetic wound healing. Although HA can be used as a practical wound dressing, therapies that integrated HA with other components such as exosomes and silver particles exhibited enhanced healing effects [16, 17]. Thus, hFDSPC-CM was mixed with HA hydrogel to endow HA with bioactivity in this study. We mainly focused on enriching HA hydrogel and endowing it with definite bioactivity compared with some previous studies that focused on controlling the mechanical strength, gelation time, and drug release of HA hydrogels [22–24].

In our current study, we successfully isolated hFDSPCs from human foreskin tissue. They exhibit the characteristics of MSCs. Although some extracellular vesicles and proteins were removed in the process of preparing condensed CM (some large extracellular vesicles larger than 220 μm have been removed in the process of filtration cell supernatants through a 0.22-μm filter; some small extracellular vesicles and protein have been removed in the process of centrifugation with a cut-off value of 10 kDa), our data revealed that hFDSPC-CM+HA accelerated cutaneous wound healing at a faster rate than did the hADSC-CM+HA treatment. The underlying mechanisms might promote epidermal proliferation and migration, acceleration of dermis closure and dermal collagen regeneration and remodelling, and inhibition of excessive inflammation.

The proliferative phase of wound healing is usually characterised by cell proliferation and migration in epidermal and dermal layers [25]. Keratinocyte migration from the wound edge is a crucial step in the re-epithelisation of cutaneous wounds. Upon injury, keratinocytes migrate over the injured dermis to re-epithelialise the damaged tissue and restore the epidermal barrier [25]. However, in a diabetic wound, the abnormal keratinocytes with obtuse migration and delayed proliferation lead to epidermis thickening at the wound edge, resulting in non-healing [26]. In the present study, complete re-epithelisation in wound tissue was observed in the hFDSPC-CM + HA-treated group, compared to the discontinuous and thickening epidermis in the other groups (Fig. 4a, b). To explain the ideal result of hFDSPC-CM + HA-treated group we observed, the in vitro assays showed hFDSPC-CM exhibited better effect to promote HaCaT proliferation and migration than did hADSC-CM (Fig. 6a, c, and d; Fig. 7a, b).

In addition to epidermal cells, fibroblasts in dermis also play an important role in skin wound healing. The rapid proliferation and migration of fibroblasts after injury determine the contraction and closure of the wound bed and the integrity of the newly formed dermis. However, fibroblast ability to proliferate and migrate is impaired in diabetic wounds compared with those in uninjured skin [27, 28]. In vivo, a complete and well-organised structure was observed in the hFDSPC-CM-treated wound tissue, but in other groups, the dermal tissue was relatively loose and irregular with numerous clots (Fig. 5a). To confirm the effect of hFDSPC-CM on fibroblasts, in vitro assays proved that hFDSPC-CM-treated human fibroblasts showed more active proliferative and migratory behaviours in vitro (Fig. 6b, e, and f; Fig. 7c, d).

Moreover, fibroblasts initiate collagen synthesis after migration to the wound site and are responsible for ECM deposition and remodelling. Wound ECM not only provides a support structure that facilitates cell migration, cell differentiation, and wound healing, but also serves as a reservoir for growth factors and mediates cell-cell, cell-matrix, and matrix-protein interactions [29, 30]. In the diabetic wound, the structure and function of ECM are considerably damaged by fibroblast dysfunction, abnormities in protein deposition, degradation, and remodelling [30]. Skin biopsies from diabetic patients exhibit lower expression of COL 1 and 3 [31], and the ratio of collagen I/III, which is closely related to ECM tensile strength, is also reduced [32]. Further, ECM in diabetic wounds exhibits anomalous structure, characterised by increased interstitial space between collagen fibres [33].

Surprisingly, our study demonstrated that hFDSPC-CM displayed a significant advantage in this process. The differentiation of fibroblasts into myofibroblasts is very important in collagen synthesis and deposition. Myofibroblasts reportedly secrete more collagen molecules, especially COL 1, thereby inducing wound contraction [34]. In the present study, a higher expression ratio of collagen I/III was observed in the hFDSPC-CM + HA-treated group rather than the HA group, illustrating that hFDSPC-CM promoted ECM maturation and remodelling (Fig. 5b, d). Meanwhile, hFDSPC-CM-treated human fibroblasts expressed more α-SMA, a marker of myofibroblasts [35] (Fig 8d, i, and j), explaining why these cells were more active in collagen secretion and deposition (Figs. 5 and 8). More evidence was found in the expression of matrix metalloproteinases, which are responsible for ECM degradation and remodelling [36] (Fig. 8e, f).

TGF-β1 is a known inducer of fibroblast differentiation, and the TGF-β/ Smad signalling pathway plays a key role in promotion of synthesis, deposition, and organisation of collagen in wound healing [21, 35, 37]. We speculated whether the TGF-β/ Smad pathway also played an important role in the regulatory effect of hFDSPC-CM. Thus, we detected TGF-β1 transcript expression levels and extracellular secretion. Our results indicated that hFDSPC-CM-treated fibroblasts expressed more TGF-β1 than did both the control group and the hADSC-CM group (Fig. 8g, h). Western blot results further supported that TGF-β/Smad signalling pathways were involved in this process (Fig. 8k). It is worth noting that excessive activation of TGF-β/Smad signalling pathways and myofibroblasts is involved in pathological scar formation [38, 39]. However, it is more likely that their activation is a physiological process in wound healing, considering that this process has been widely reported previously [40, 41]. Nevertheless, we cannot rule out the possibility that hFDSPC-CM activates pathological scar formation. It is worth studying the effect of hFDSPC-CM on scar formation in the future in a rabbit ear scar model rather than mouse skin wound healing model, considering that no obvious scar forms on mouse skin [42].

Prolonged inflammation is observed in diabetic wounds with the persistence of excessive macrophages at the wound sites [43, 44]. It has been reported that suppressing inflammation may increase collagen production and re-epithelialisation [45]. Our results showed that hFDSPC-CM treatment significantly reduced macrophage infiltration (CD68-positive cells) and promoted macrophage polarisation from M1 (CD86-positive cells) to M2 (CD206-positive cells) type macrophages in diabetic mouse wounds (Fig. 4e–j), suggesting its effect on anti-inflammation and promotion of skin regeneration in wound healing. This may also promote re-epithelialisation and collagen synthesis and remodelling.

Another essential stage of wound healing is adequate perfusion through capillary networks, which is facilitated by angiogenesis [46]. In our study, hFDSPC-CM showed a similar ability to hADSC-CM in promotion of neovascularisation in vitro and in vivo (Fig. 4c, d; Fig. S2). These results also confirmed the effect of hFDSPC-CM in promoting skin wound healing.

In summary, hFDSPCs may present a novel alternative source for therapeutic use in diabetic wound healing. hFDSPC-CM endowed HA with good bioactivity for skin wound healing in multiple ways. It presents significant advantages in reducing inflammation, collagen synthesis, and remodelling compared with hFDSPC-CM. Although we observed the effect of hFDSPC-CM on diabetic wound healing, the precise active ingredients within it need to be further confirmed and the related regulatory mechanisms should be further explored. In addition, the immunogenicity and safety assays in this study are insufficient, and we will discuss these contents in detail in future studies for clinical application.

## Conclusions

In comparison with hADSC-CM, hFDSPC-CM endowed HA with superior wound healing effects by accelerating diabetic wound closure and promoting wound closure rate. The underlying mechanism may contribute to promoting proliferation and migration of epidermal cells with fibroblasts, thus leading to ECM deposition and remodelling. Decreased inflammation may be attributed to the above phenomenon.

## Supplementary Information

**Additional file 1: Fig. S1.** Identification of the characteristics of hADSCs. A trilineage-induced differentiation experiment to confirm multiple differentiation potential. The cells at passage 2 were used in all experiments. The osteogenesis potential was examined using alizarin red staining, bar = 100 μm (a). Adipogenesis was analysed using oil red O staining, bar = 50 μm (b). Chondrogenesis was assessed using alcian blue staining, bar = 200 μm (c). Immunophenotyping of hADSCs was characterised using flow cytometry analysis (d). hADSCs were analysed for expression of the following markers: CD19 (1.17% ± 0.69%), CD34 (0.83% ± 0.35%), CD11b (1.50% ± 0.86%), CD45 (0.99% ± 0.54%), HLA-DR (1.20% ± 0.45%), CD73 (97.97% ± 1.19%), CD90 (97.03% ± 1.45%), and CD105 (97.04% ± 1.12%). Data are shown as means ± SD, *n* = 4. **Fig. S2.** Tube formation assay of HUVECs in vivo. (a) HUVECs treated with hADSC-CM or hFDSPC-CM (20 μg/mL) were evaluated after 6 h, and the PBS treatment was used in the control group, bar = 25 μm. (b) Assessment of number of branches in each group. (c) Quantification of mean tube length. Data are shown as means ± SD; n = 4 **p < 0.01, ***p < 0.001. **Fig. S3.** The test of hydrogel adhesion. The hydrogel sticked to the walls of the bottle without sliding down.

## Data Availability

The datasets generated during and/or analysed during the current study are available from the corresponding author on reasonable request.

## Abbreviations

DPSCs: Dermis-derived progenitor/stem cells
CM: Conditioned medium
HA: Hyaluronic acid
hFDSPCs: Human foreskin-derived dermal stem/progenitor cells
hFDSPC-CM: Human foreskin-derived dermal stem/progenitor cell-conditioned medium
hADSCs: Human adipose-derived stem cells
hADSC-CM: Human adipose-derived stem cell-conditioned medium
HUVECs: Human umbilical vein endothelial cells
ECM: Extracellular matrix
COL 1: Collagen I
COL 3: Collagen III
FN: Fibronectin
MMP-1: Matrix metalloproteinases 1
MMP-3: Matrix metalloproteinases 3
TGF-β1: Transforming growth factor-beta 1
MSCs: Mesenchymal stem cells
EVs: Extracellular vesicles
HaCaT: Human immortal keratinocyte cells
HUVECs: Human vascular endothelial cells
HE: Haematoxylin and eosin
