# Supplementary material for: Human foreskin-derived dermal stem/progenitor cell-conditioned medium combined with hyaluronic acid promotes extracellular matrix regeneration in diabetic wounds
Source: Stem Cell Res Ther. 2021 Jan 9;12:49. doi: 10.1186/s13287-020-02116-5 (PMC7796620; doi:10.1186/s13287-020-02116-5)
Supplement: Supplementary file 1 — Additional file 1: Fig. S1. Identification of the characteristics of hADSCs. A trilineage-induced differentiation experiment to confirm multiple differentiation potential. The cells at passage 2 were used in all experiments. The osteogenesis potential was examined using alizarin red staining, bar = 100 μm (a). Adipogenesis was analysed using oil red O staining, bar = 50 μm (b). Chondrogenesis was assessed using alcian blue staining, bar = 200 μm (c). Immunophenotyping of hADSCs was characterised using flow cytometry analysis (d). hADSCs were analysed for expression of the following markers: CD19 (1.17% ± 0.69%), CD34 (0.83% ± 0.35%), CD11b (1.50% ± 0.86%), CD45 (0.99% ± 0.54%), HLA-DR (1.20% ± 0.45%), CD73 (97.97% ± 1.19%), CD90 (97.03% ± 1.45%), and CD105 (97.04% ± 1.12%). Data are shown as means ± SD, n = 4. Fig. S2. Tube formation assay of HUVECs in vivo. (a) HUVECs treated with hADSC-CM or hFDSPC-CM (20 μg/mL) were evaluated after 6 h, and the PBS treatment was used in the control group, bar = 25 μm. (b) Assessment of number of branches in each group. (c) Quantification of mean tube length. Data are shown as means ± SD; n = 4 **p < 0.01, ***p < 0.001. Fig. S3. The test of hydrogel adhesion. The hydrogel sticked to the walls of the bottle without sliding down. [file 13287_2020_2116_MOESM1_ESM.docx]

# supplementary materials

# Human foreskin-derived dermal stem/progenitor cells conditioned medium combined with [hyaluronic](javascript:;) [acid](javascript:;) promotes extracellular matrix regeneration in diabetic wounds

**Authors:** Yu Xin^1*^, Peng Xu^1,2*^, Xiangsheng Wang^1,2^, Yusheng Chen^1^, Zheng Zhang^1#^, Yixin Zhang^1#^

**Affiliations:**

1. Department of Plastic and Reconstructive Surgery, Shanghai 9th People's Hospital, Shanghai Jiao Tong University School of Medicine, 639 Zhi Zao Ju Road, Shanghai, 200011, China
2. Shanghai Tissue Engineering Key Laboratory, Shanghai Jiao Tong University School of Medicine, Shanghai 200011, China

* ***These authors contributed equally to this study.***

***^#^ Corresponding authors:***

1. Zheng Zhang, MD; PhD

Email: zhangzheng958@163.com

2. Yixin Zhang, MD; PhD

Email: zhangyixin6688@163.com

**Fig. S1.**

#
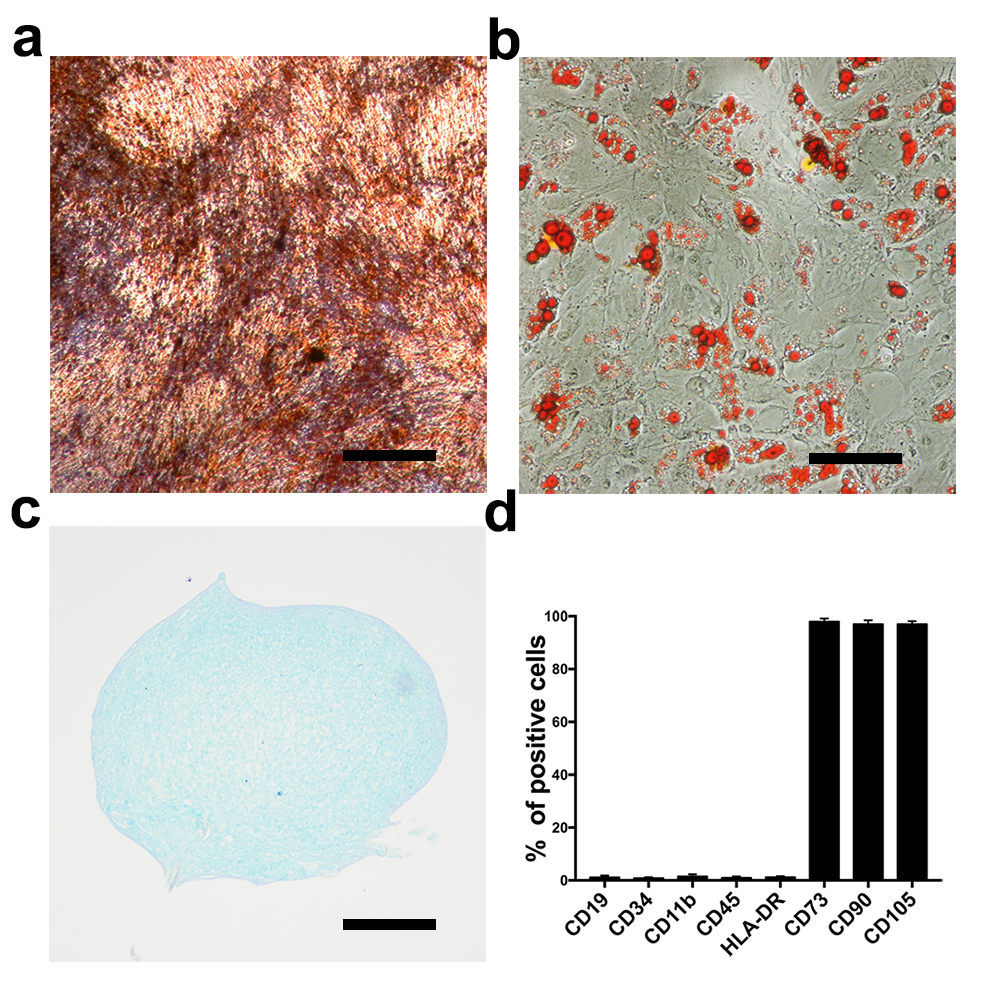


**Fig. S1.** Identification of the characteristics of hADSCs. A trilineage-induced differentiation experiment to confirm multiple differentiation potential. The cells at passage 2 were used in all experiments. The osteogenesis potential was examined using alizarin red staining, bar = 100 μm (a). Adipogenesis was analysed using oil red O staining, bar = 50 μm (b). Chondrogenesis was assessed using alcian blue staining, bar = 200 μm (c). Immunophenotyping of hADSCs was characterised using flow cytometry analysis (d). hADSCs were analysed for expression of the following markers: CD19 (1.17% ± 0.69%), CD34 (0.83% ± 0.35%), CD11b (1.50% ± 0.86%), CD45 (0.99% ± 0.54%), HLA-DR (1.20% ± 0.45%), CD73 (97.97% ± 1.19%), CD90 (97.03% ± 1.45%), and CD105 (97.04% ± 1.12%). Data are shown as means ± SD, n = 4.

**Fig. S2**


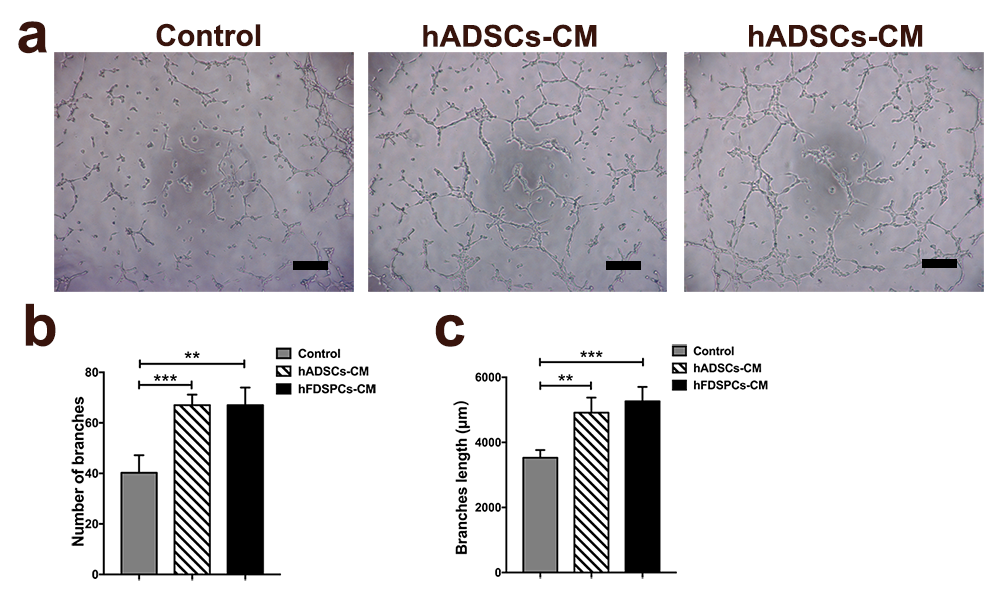
**.**

**Fig. S2.** Tube formation assay of HUVECs *in vivo*. (a) HUVECs treated with hADSCs-CM or hFDSPC-CM (20 μg/mL) were evaluated after 6 h, and the PBS treatment was used in the control group, bar = 25 μm. (b) Assessment of number of branches in each group. (c) Quantification of mean tube length. Data are shown as means ± SD; n = 4 ***p* < 0.01, ****p* < 0.001.

**Fig. S3**


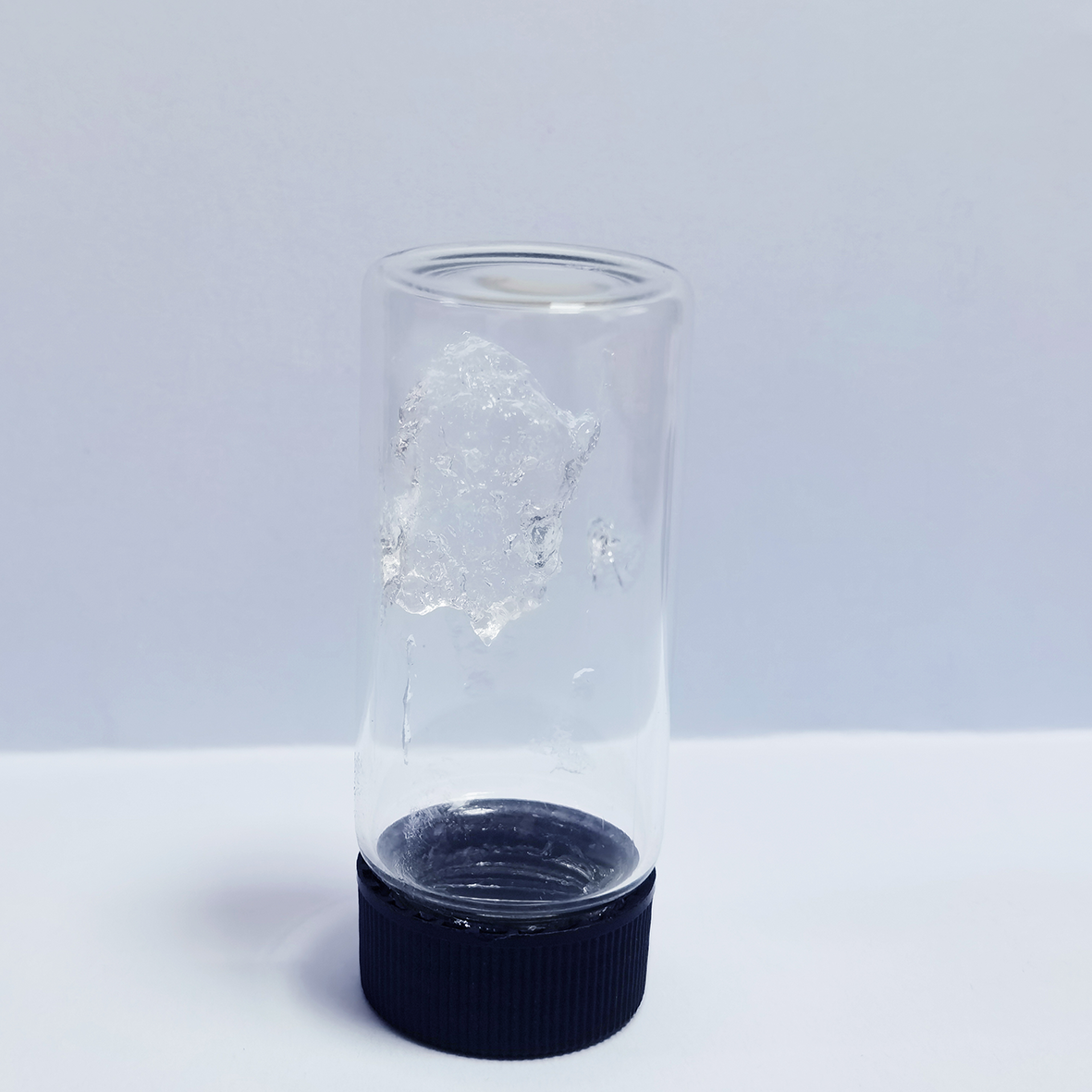


**Fig. S3.** The test of hydrogel adhesion. The hydrogel sticked to the walls of the bottle without sliding down.
